# Increased UBE2L6 regulated by type 1 interferon as potential marker in TB

**DOI:** 10.1111/jcmm.17046

**Published:** 2021-11-12

**Authors:** Jiao Gao, Chonghui Li, Wenjing Li, Haotian Chen, Yurong Fu, Zhengjun Yi

**Affiliations:** ^1^ School of Medical Laboratory Weifang Medical University Weifang China; ^2^ School of Basic Medicine Weifang Medical University Weifang China

**Keywords:** tuberculosis, biomarker, UBE2L6, miR‐146a‐5p

## Abstract

The aim of this study is to identify potential biomarker of tuberculosis (TB) and determine its function. Differentially expressed mRNAs(DEGs) were selected from a blood database GSE101805, and then, 30 key genes were screened using STING, Cytoscape and further functionally enriched. We then found that only 6 of 13 genes related to ubiquitination (the first in the functional enrichment) were increased significantly. ROC analysis showed that UBE2L6, among 6 genes, had the highest diagnostic value, and then, we found that it also had mild value in differential diagnosis. Moreover, our analysis showed that UBE2L6 may be upregulated by type I interferon, which was further confirmed by us. In addition, we also found that UBE2L6 inhibits the apoptosis of *Mycobacterium tuberculosis*(Mtb)infected macrophages. Subsequently, we discovered that miR‐146a‐5p, which may target UBE2L6, is reduced in peripheral blood mononuclear cells (PBMC) and plasma of TB, and it also had certain diagnostic efficiency(AUC=0.791). In brief, we demonstrated that UBE2L6 as well as miR‐146a‐5p is a potential biomarker for TB and UBE2L6,which may also plays important role in TB by, at least, modulating Mtb‐infected macrophage apoptosis.

## 1 INTRODUCTION

Tuberculosis(TB) is a chronic infectious disease caused by infection of *Mycobacterium tuberculosis*(Mtb). More than 4,000 people die of TB and nearly 30,000 become infected with this disease every day. TB remains the world's deadliest infectious disease killer.^1^ Mtb can invade susceptible organisms and cause TB through respiratory tract, digestive tract or skin injury, among which respiratory tract is the most common.

Although the immune response of Mtb infection is mainly concentrated in the lungs, its pathological state can be reflected in the peripheral blood through circulating immune cells.^2^ Whole‐blood transcriptomics provide important information about the host immune response and are an important tool for identifying potential markers of infection. RNA expression analysis has become a powerful tool for understanding disease biology.^3^ Many diseases, such as cancer^4^, ^5^ and infectious diseases,^6^ including TB,^7^ are associated with specific transcriptional profiles in blood or tissue. Differentially expressed genes (DEGs) screened from blood transcriptome can help us know the pathogenesis at the molecular level, and further service for the diagnosis and treatment of diseases.

At present, TB diagnosis is mostly based on epidemiological characteristics, combined with microbiological tests and chest X‐rays. Respiratory symptoms such as cough and expectoration and systemic symptoms such as low fever, night sweats, emaciation and weakness will be noticed; however, some patients have no obvious clinical manifestations. Although there are accepted methods for diagnosis of TB, they have well‐known disadvantages, such as insufficient sensitivity (sputum smear) and long turnaround time (culture for 4 to 8 weeks) for the results.^8^ In addition, Xpert provide essential information on rifampin resistance, it is less sensitive than TB sputum culture and cannot distinguish Non‐tuberculous mycobacterium (NTM) infections.^9^ Furthermore, the Xpert assay has limited performance for TB smear negatives and paediatric specimens (around 67% sensitivity).^10^ Specific response networks were constructed by mapping disease genome‐wide expression. The DEGs screened out can be used as diagnostic and differential diagnostic markers of disease and indicators of disease change.

**FIGURE 1:**
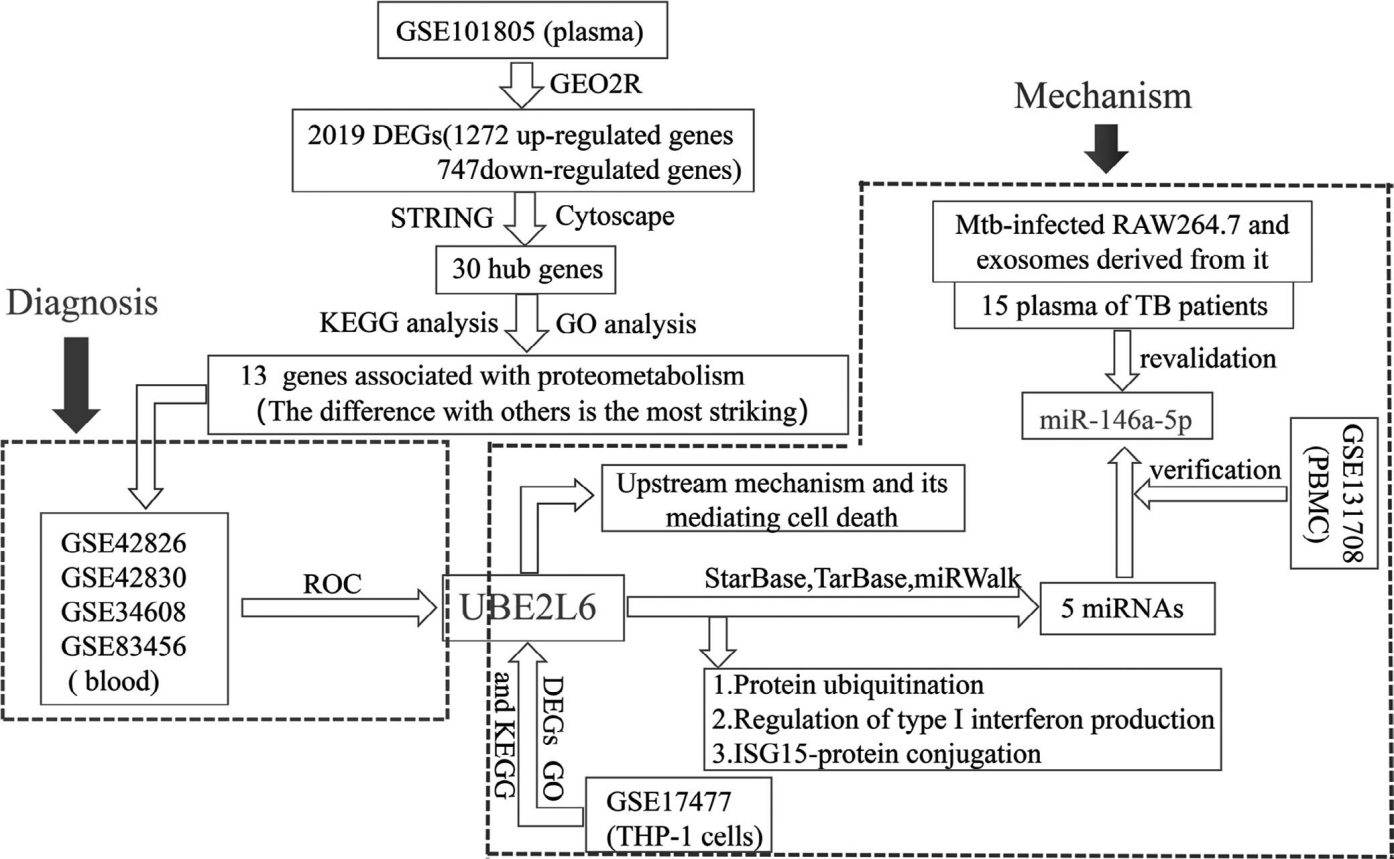
The flowchart of the study

The purpose of this study was to identify potential biomarker in blood of TB and determine its function in Mtb‐infected macrophages. Bioinformatics methods were used for data analysis to identify the most influential genes in the response network of TB, and then, functional enrichment was conducted, followed by validation, diagnostic and differential diagnostic value analysis. Subsequently, DEGs in the THP‐1 cells were enriched and the role of the final hub gene was determined, its function was further studied in RAW264.7 cells. These will provide new thoughts for the diagnosis and treatment of TB. The flowchart of the study was shown in Figure 1.

## 2 MATERIALS AND METHODS

### 2.1 Data processing and identification of DEGs

The microarray data in this study were retrieved and downloaded from the NCBI gene expression omnibus(GEO) database(http://www.ncbi.nlm.nih.gov/geo/) using keywords ‘TB’, ‘Mtb’ and ‘Human’. After comparison, the plasma source data set GSE101805 was selected, its microarray platform is GPL16956 whose chip probes include LNCRNAs and mRNAs. The corresponding table of probe and gene was obtained by BLAST reannotation. The original data had been normalized. The GEO2R online tool was used to analyse the data, and DEGs were selected according to the following criteria:*p*‐value<0.05,|logFC|≥2.

### 2.2 Screening of hub genes and function prediction

STRING software was used to obtain the protein interaction network (PPI) of DEGs, and then, import the analysis results into Cytoscape V.3.7. Cytohubba and MCODE plug‐ins were used to screen key subnetworks and key protein modules. The hub genes were obtained by crossing the first 30 Cytohubba genes and the cluster with the highest score of MCODE. DAVID and FUNRICH software were used for Gene Ontology(GO) and Kyoto Encyclopedia of Genes and Genomes(KEGG) analysis of the hub genes.

### 2.3 GEO database validation and ROC analysis of hub genes

We selected three TB‐related databases (GSE34608,GSE42826,GSE42830) to verify the hub genes. Three databases include blood samples of 35 TB patients and 50 healthy controls (HC). GSE83456 is used for differential diagnostic analysis, and it include 61 HC, 47 human with extra‐pulmonary TB(EPTB), 45 human with pulmonary TB(PTB), 49 human with sarcoid. ROC curves were analysed by SPSS Statistics 26 software.

### 2.4 Functional enrichment of DEGs in cells

In order to explore the expression and function of the key hub gene in Mtb‐infected macrophages, we used the GSE17477 gene data set derived from Mtb‐infected THP‐1 cells for subsequent analysis. The GEO2R online tool is used to process the data and filter the DEGs according to the same criteria as above. Importing them into DAVID and FUNRICH software for GO and KEGG analysis.

### 2.5 miRNAs prediction of key hub gene and validation

miRWalk, StarBase and TarBase were used to predict the miRNAs which may target key hub gene, and the online software Bioxinren was used to draw the Venn diagram. We selected a PBMC database GSE131708, which included 4 patients with TB meningitis and 4 healthy individuals, to verify the reliability of the predicted miRNAs in TB. The raw data have been normalized.

### 2.6 Clinical samples collection and Quantitative reverse transcription‐polymerase chain reaction (qRT‐PCR)

A total of 30 plasma samples (15 TB and 15 HC) were collected from Weifang Second People's Hospital and Weifang Medical University, and there was no significant difference in age and gender between them. Informed consent has been obtained from all participants. None of the patients were received anti‐TB treatment prior to sample collection. TB patients were diagnosed by sputum culture and smear positivity, if both culture and smear is negative, the first 3 items of the following are met simultaneously or one of the item 4 and item 5 of the following can be confirmed,(Ⅰ) Typical clinical symptoms of TB (cough, expectoration, hemoptysis) and typical chest imaging findings. (Ⅱ) TB serological auxiliary test was positive. (Ⅲ) The sputum sample positive for PCR. (Ⅳ) Lung tissue pathology confirmed TB. (Ⅴ) Bronchoalveolar lavage proved positive for TB. People with HIV, HBV and severe autoimmune diseases were excluded. Relevant research was approved by the ethics committee of Weifang Medical University.

Total RNA was extracted from each sample using TRlzol‐LS, and its concentration and purity were assessed by K5800 Micro‐spectrophotometer (Kaiao). The reverse transcription was conducted using PrimeScript™ 1st Strand cDNA Synthesis Kit (Takara) at 42℃ for 60 minutes and then at 95℃ for 5 minutes. Next, based on LightCycler^®^ 480 II real‐time PCR system (Roche), PCR was performed with SYBR^®^ Premix Ex Taq™ Kit (Takara) at the temperature of 95℃ for 60 s, followed by 40 cycles with the temperature of 95℃ for 60 s, 60℃ for 60 s. U6 was applied as internal controls. The 2^−ΔΔCt^ method was utilized to determine the relative expression of the selected miRNA between case and control.

### 2.7 Culture of RAW264.7 cells and Mtb infection

4‐6ml RPMI 1640 medium containing 10% foetal bovine serum (FBS) was added into the cell culture flask and cultured in a cell incubator at 37℃ and 5% CO_2_. When the cells grew to the logarithmic growth phase, open the cell culture flask, pour out the old medium, wash with PBS and add 2 ml trypsin. The cells were observed under a microscope, after cells became round, fresh medium was added to stop digestion. Next, the cells were repeatedly blown with a pasteurian tube, then the cell suspension was collected, centrifuged at 800 rpm for 2 min, and the cells were resuspended for subculture and planking.

According to the density of 6 × 10^5^/ well, the cells were inoculated in six‐well plate. When cells adhered to the wall and grew exuberantly, a small amount of medium was discarded, bacterial liquid was added in proportion, and grouping was marked. After 4h, fresh medium was replaced for further culture, which was marked as infection 0.

### 2.8 IFNAR block, siRNA transfection and Propidium Iodide (PI)/Hoechst 33342 Double Staining

IFNAR neutralizing antibody (Leinco Technologies, USA) was added to cell culture plates at a concentration of 10μg/ ml for 2 h prior to Mtb infection. Then, total RNA was extracted from cells infected 24 h with Mtb, followed by reverse transcription and amplification.

Three siRNA for the final gene were synthesized by Sangon Biotech. The sense and antisense strands of UBE2L6 siRNA1 are 5′‐CCA GGG AGU AUC CAU UCA ATT‐3′ and 5′‐UUG AAU GGA UAC UCC CUG GTT‐3′. The sense and antisense strands of UBE2L6 siRNA2 are 5′‐GCU GGUNGAG UAA ACC GAA UTT‐3′ and 5′‐AUU CGG UUU ACU CAC CAG CTT‐3′. The sense and antisense strands of UBE2L6 siRNA3 are 5′‐GCC CUC UUA AUU CUG UUC UTT‐3′ and 5′‐AGA ACA GAA UUA AGA GGG CTT‐3′. The sense and antisense strands of negative control (NC) siRNA are 5′‐UUC UCC GAA CGU GUC ACG UTT‐3′ and 5′‐ACG UGA CAC GUU CGG AGA ATT‐3′. After transfection with Lip2000 (Thermo Fisher Scientific) in the ratio of 1:1, we screened out the one with the most interference effect for subsequent experiments.

Twenty‐four hours after transfection, Mtb infection was carried out at a ratio of 1:10. After 24 h of infection, the supernatant was discarded and 500μl PBS was added, followed by 5μl Hoechst and 5μl PI staining solution. After mixing, the cells were incubated at 4 degrees for 20 min. Finally, fluorescence microscope was used to observe.

### 2.9 Extraction and identification of exosomes

FBS was inactivated and centrifuged at 120,000 g for 16 h, 70% supernatant of it was added to RPMI1640 medium. The medium containing 10% FBS was prepared and filtered by 0.22μm microporous membrane. Supernatants of cells from the infected group and the uninfected group for 48 h were at 4℃ for 300 g, 10 min, the supernatant was taken. The supernatant was further centrifuged at 4℃ for 20 min at 2000 g, and then, the supernatant was removed and centrifugation was continued at 4℃ for 10,000 g for 30 min, and the supernatant was removed to hard overspeed centrifugal tubes for centrifugation at 4℃, 100,000 g for 70 min. Carefully discard supernatant, do not touch precipitation. PBS was added to resuspend, the previous centrifugation process was repeated, supernatant was discarded again, and the precipitation was resuspended with 100‐500μl PBS and filtered with 0.22μm microporous membrane.

Transmission electron microscopy (TEM) was used to identify the microstructure of exosomes. 15μl exosomes suspension was added to the copper net and precipitated for 2 min. The floating liquid on the edge of the copper net was carefully sucked away. 10μl uranium dioxide acetate was dropped into the copper net, dyed for 1 min, and the floating liquid was also absorbed. After drying at room temperature, the samples were observed under TEM.

Nanoparticle tracking analysis (NTA) was used to identify exosome diameter abundance. 100uL exosomes suspension was added to sterile filtered PBS and diluted to 1mL, then added to colorimetric dish. The diameter abundance of exosome samples was measured using NTA size model.

Western blot (WB): The cells were lysed using a cell lysis buffer (SolarBio, China) according to the instructions, and the protein concentration was determined using the BCA protein quantification kit (Beyotime, China). The protein samples were electrophoresed in sodium dodecyl sulphate‐polyacrylamide gel electrophoresis (SDS‐PAGE), and transferred to PVDF membrane, blocked with 5% skim milk. After incubation with corresponding primary antibody, it was incubated with HRP‐labelled goat anti‐rabbit IgG secondary antibody.

### 2.10 Reverse transcription‐polymerase chain reaction (RT‐PCR)

Total RNA from cells and exosomes was extracted by Trizol. The concentration and purity of RNA were then measured by K5800 differential photometer (KAIAO). The reverse transcriptional condition of miRNA is 42℃ for 60 min, then 95℃ for 5 min, and finally 4℃ for 15 min, followed by amplification on a gradient PCR amplification instrument (Thermo Fisher) at 94℃ for 15 s, 58℃ for 25 s, 72℃ for 25 s. The reverse transcriptional condition of the screened gene is 42℃ for 45 min, then 95℃ for 5min, followed by amplification at 94℃ for 30 s, 63℃ for 30 s, 72℃ for 60 s. The annealing temperature of U6 and tubulin is 60℃ and 58℃, other conditions are the same.

### 2.11 Data analysis

Statistical analysis was performed using IBM SPSS Statistics 26 software. Statistical significance of differences between and among groups was assessed using the t test. Plot uses mean with SD. Box diagram was drawn using originPro9. 1. Significant differences are indicated as follows: * *p *< 0.05,* * *p *< 0.01,* * *p < 0.001.

## 3 RESULTS

### 3.1 DEGs and hub genes were screened

**FIGURE 2:**
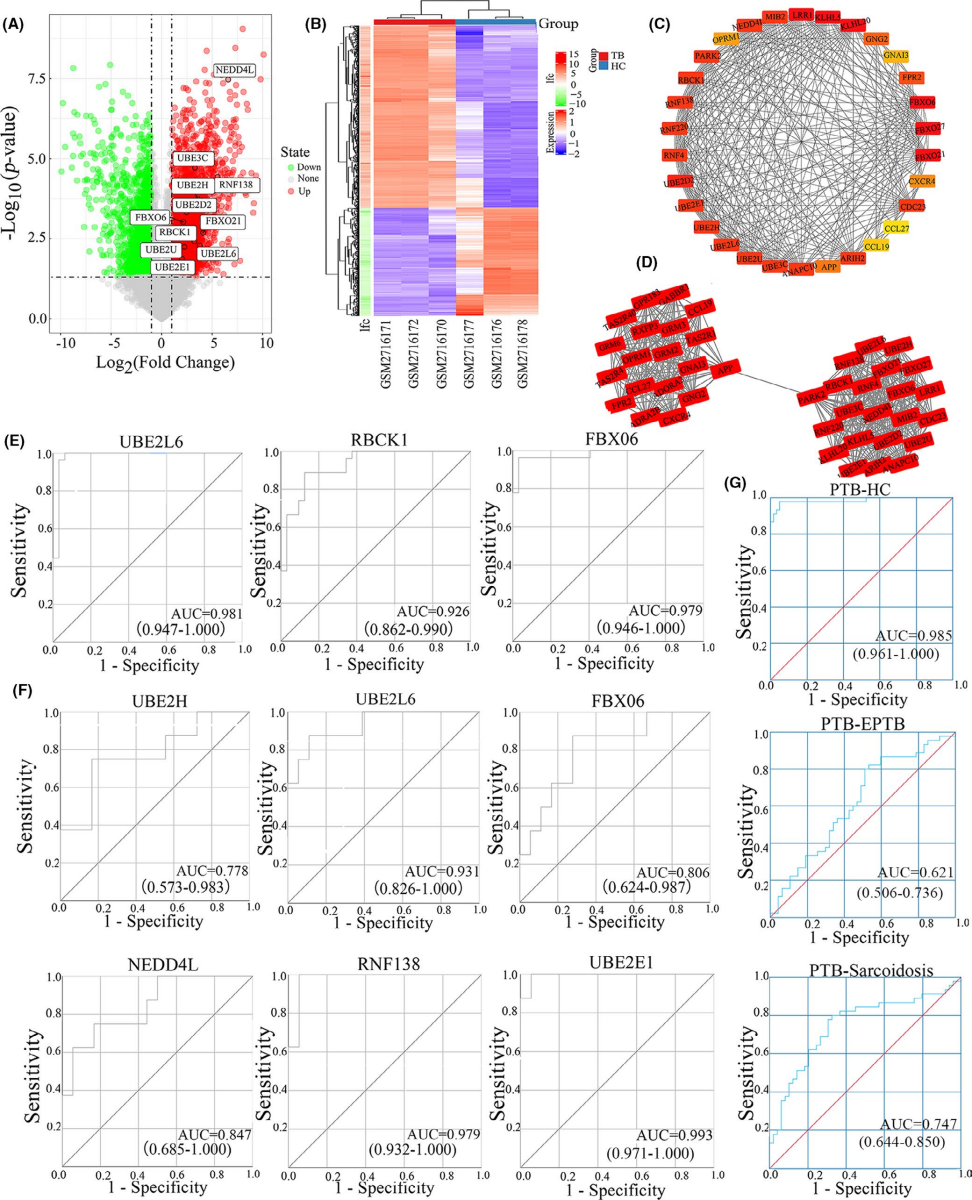
DEGs and hub genes screened Volcanic diagram of all mRNAs in GSE101805 database(A), Red: upregulated mRNAs. Green: downregulated mRNAs. Grey: normally expressed mRNAs. Hierarchical clustering analysis of 2,019 DEGs(B), each row represents one mRNA, and each column represents one sample. lfc:Log fold change. The first 30 hub genes found on Cytohubba plug‐ins(C), The darker the colour, the higher the score. The gene cluster with the highest score selected by the MCODE plug‐in(D).ROC curves of 4 hub genes in GSE42826 and GSE42830(E). ROC curves of 6 hub genes in GSE34608(F). Evaluation of differential diagnostic value of UBE2L6 in GSE83456(G)AUC: Area under the curve

Through GEO2R analysis of the mRNA data set of healthy people and TB in GSE101805, we identified 2,019 DEGs, including 1272 upregulated genes and 747 downregulated genes (Figure 2A). The expression level of DEGs is visualized in the form of clustering heat map (Figure 2B).

In view of the sample type and research purpose, only the upregulated DEGs were selected for the follow‐up study. We uploaded these genes to STRING software to build the protein interaction (PPI) network, which consists of 1249 nodes and 4837 edges. To find the genes that play central roles, we imported the built PPI network into Cytoscape software, whose Cytohubba and MCODE plug‐ins were used to mine important nodes in the network. The first 30 hub genes found on Cytohubba plug‐ins (Figure 2C). The gene cluster with the highest score selected by the MCODE plug‐in (Figure 2D). By combining the two algorithms, a total of 30 hub genes were screened out.

### 3.2 Functional enrichment of hub genes

In order to determine the function of the 30 hub genes mentioned above, we used DAVID and FUNRICH software to conduct GO and KEGG analysis. Results of DAVID showed that these hub genes were mainly connected with protein ubiquitination (*p *= 3.39E^−21^), protein modification by small protein conjugation (*p *= 5.60E^−20^), modification‐dependent protein catabolic process (*p *= 8.85E^−17^), etc. FUNRICH analysis results showed that they were mainly related to protein metabolism (*p *= 5.46E^−09^), regulation of cell cycle(*p *= 0.0902).

### 3.3 Validation of key hub genes and differential diagnostic value evaluation

We picked out 13 genes associated with protein ubiquitination, and they were FBXO6, FBXO21, UBE2D2,NEDD4L, UBE2L6, UBE2E1, PARK2, UBE3C, RBCK1, ARIH, RNF138, UBE2U and UBE2H. We then performed validation and diagnostic value prediction. Three peripheral blood gene data sets GSE34608, GSE42826 and GSE42830 were used for verification. GSE42826 and GSE42830 are the detection and verification sets of the GSE42834 series, they use the same platform and data processing methods, so the data of the two data sets are combined for analysis here. The genes with *p* ≥ 0. 05 and logFC<0 were excluded by GEO2R analysis, then GSE34608 database remained UBE2E1, UBE2L6, NEDD4L, RNF138, UBE2H, FBXO6 six genes, and the other two databases have UBE2L6, FBXO6, RBCK1 three genes left. We further performed ROC analysis of the above genes using the same databases (Figure 2E,F), and two analysis results showed that UBE2L6 has the highest diagnostic efficiency among these genes, its AUC, sensitivity and specificity were 0.931, 87.5%, 88.9%(95%CI:0.826–1.000) and 0.981, 100%, 93.8%(95%CI:0.947–1.000). Compared with WHO target product profile (TPP), the sensitivity and specificity of UBE2L6 could not all reach the optimal ideal state (90% sensitivity, 90% specificity), but far higher than the minimum standard (75% sensitivity, 75% specificity).^11^, ^12^, ^13^ Next, we evaluated the differential diagnostic value of UBE2L6 using GSE83456 (Figure 2G), which included PTB,EPTB and sarcoid patients, the results showed that UBE2L6 had good diagnostic value in differentiating HC from PTB (AUC:0.985, 97.8% sensitivity, 95.1% specificity, 95%CI:0.961–1.000), but it is not very good at distinguishing EPTB from PTB(AUC:0.621,8.22% sensitivity,4.68% specificity,95%CI:0.506–0,736),and PTB from sarcoidosis (AUC:0.747, 80.0% sensitivity, 67.3% specificity, 95%, CI:0.644–0.850). Except for PTB from HC, PTB from EPTB as well as PTB from sarcoidosis have good sensitivity but low specificity.

### 3.4 Bioinformatics analysis of UBE2L6 in Mtb‐infected THP‐1 cells

**FIGURE 3:**
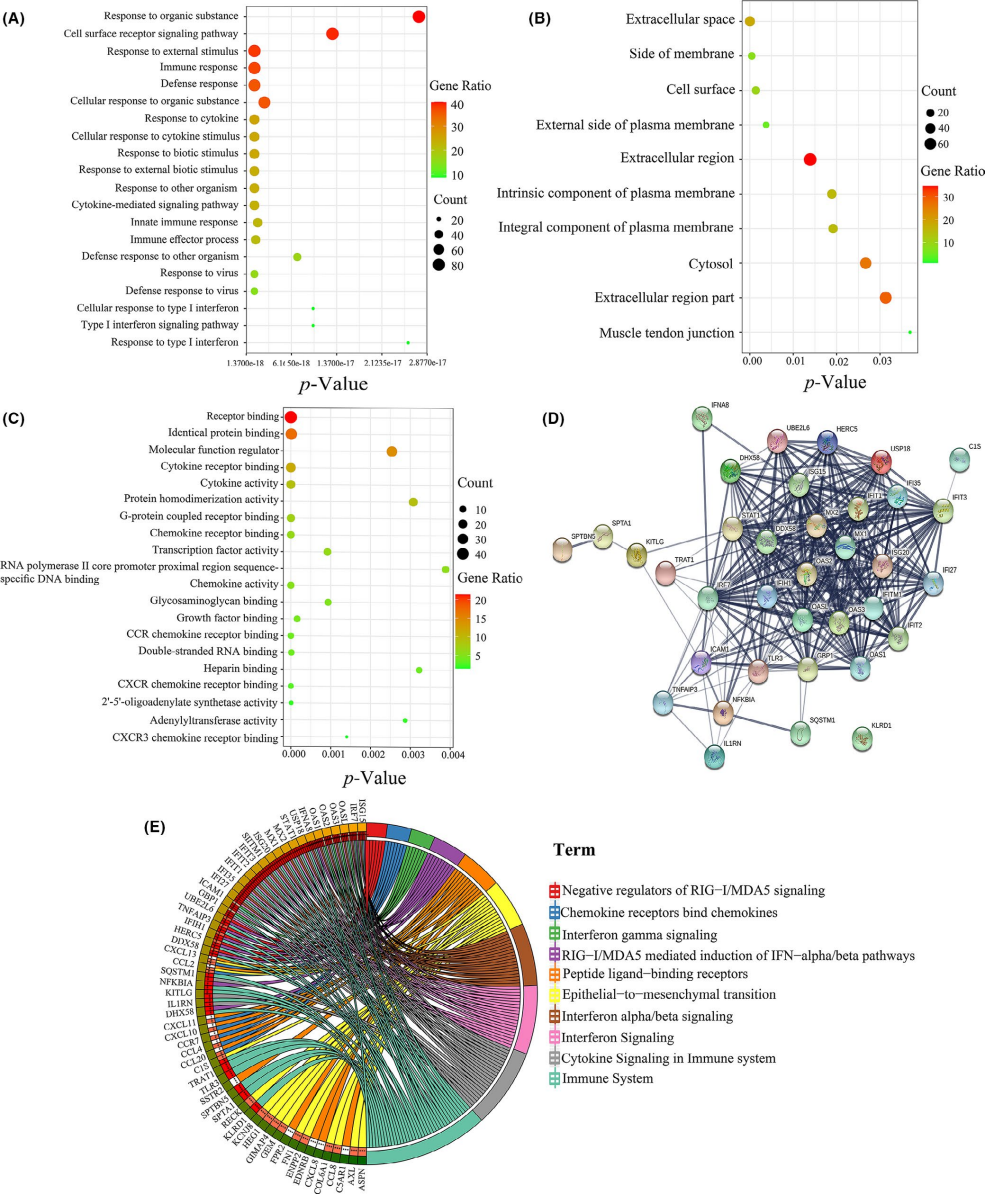
GO and KEGG analysis of DEGs. Top 20 of the BP analysis of 240 DEGs(A). CC: analysis of 240 DEGs(B). Top 20 of the MF analysis of 240 DEGs(C). Top 10 KEGG analyses of 240 DEGs(D). PPI network diagram of UBE2L6 and its related mRNAs(E). BP:Biological Process CC: Cellular Component MF: Molecular Function KEGG: Kyoto Encyclopedia of Genes and Genomes PPI: Protein‐Protein Interaction

After the diagnostic value of UBE2L6 was clarified in TB, we wanted to further explore the mechanism of UBE2L6 in Mtb‐infected macrophages. Therefore, we used the mRNA gene database GSE17477 derived from Mtb‐infected THP‐1 cells for analysis and study. GEO2R analysis results showed that *p*‐value of UBE2L6 was 7.04E^−07^, and the logFC value was 2.3163, indicating that UBE2L6 was significantly upregulated in Mtb‐infected THP‐1 cells. Next, we used DAVID and FUNRICH software to conduct GO analysis (Figure 3A‐C) and KEGG pathway prediction (Figure 3E) on 240 DEGs. The results showed that UBE2L6 was mainly located in the cytoplasm and was important for regulating cytokine production (*p *= 1.33E^−12^), type I interferon production (*p *= 5.18E^−06^), binding to ISG15 protein (*p *= 0.002) and protein modification (*p *= 0.0077). KEGG analysis showed that it was mainly involved in the immune system (*p *= 2.28E^−11^) and the induction of IFN‐alpha/beta pathway mediated by RIG‐I/MDA5 (*p *= 7.95E^−07^). In short, UBE2L6 was not only involved in protein ubiquitination, but also correlated with ISGylation of proteins and other innate immune pathways. UBE2L6 and the predicted genes related to it were imported into STRING software to draw PPI network (Figure 3D), which consisted of 36 nodes and 309 edges.

### 3.5 UBE2L6 induced by type I interferon in RAW264.7 cells during Mtb infection and then inhibits apoptosis

**FIGURE 4:**
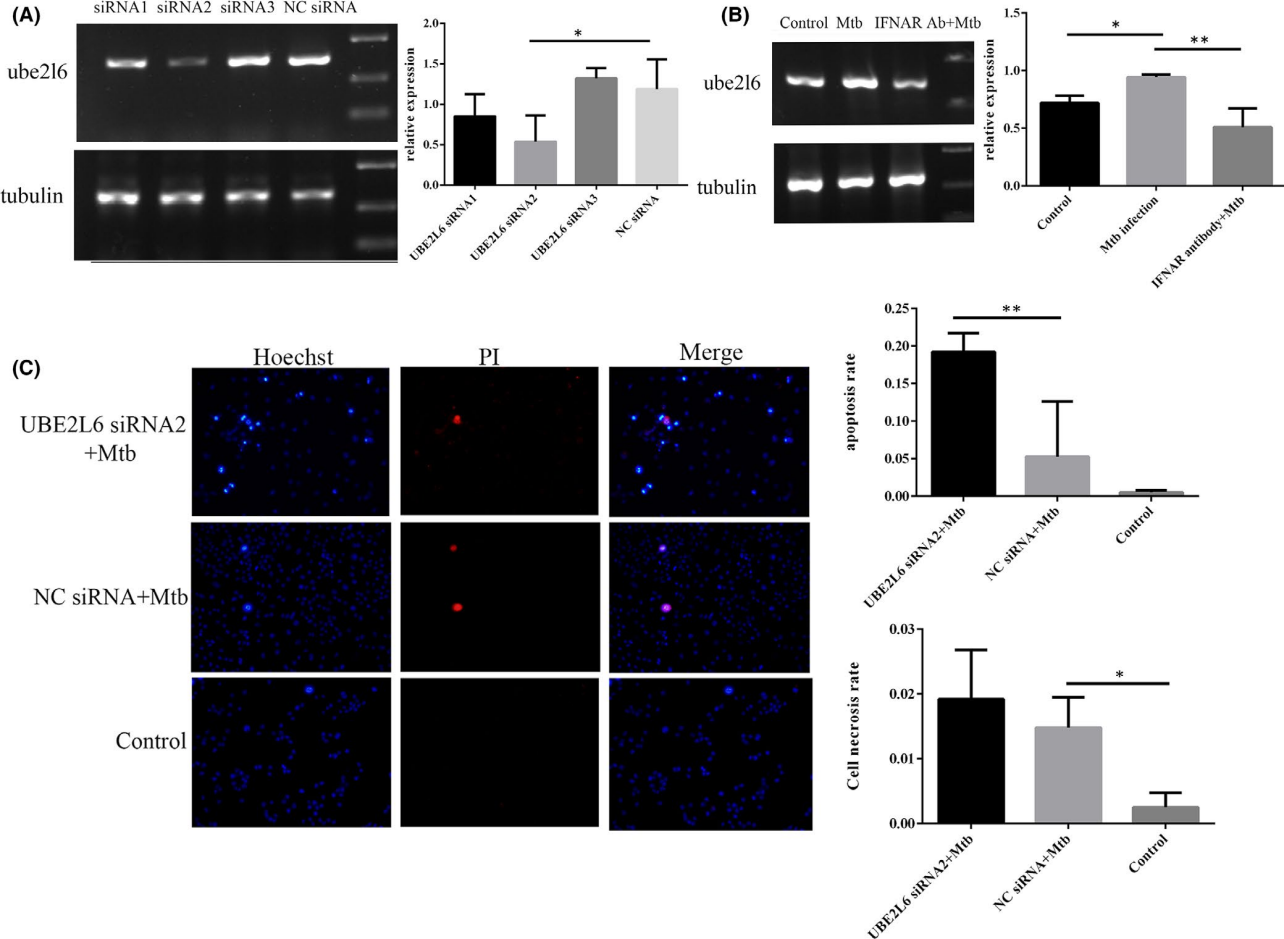
Type I interferon induced UBE2L6 which inhibits apoptosis. Most effective siRNA screening(A). Effect of type I interferon on UBE2L6(B). PI/Hoechst 33342 Double Staining(C) Normal cells showed weak red fluorescence +weak blue fluorescence, apoptotic cells showed weak red fluorescence +strong blue fluorescence, necrotic cells showed strong red fluorescence +strong blue fluorescence.*:*p* < 0. 05 **:*p *< 0. 01***:*p *< 0. 001

On the basis of the above bioinformatics analysis, we want to further explore the expression of UBE2L6 in RAW264.7 cells and its relationship with type I interferon, and explore its effect on cells. Our results showed that UBE2L6 was significantly increased (*p *= 0.037) in RAW264.7 cells after Mtb infection(Figure 4B). Neutralizing IFNAR with antibodies to inhibit type I interferon pathways, and then infecting cells, we found that UBE2L6 was down (Figure 4B). This suggests that UBE2L6 can be induced by type I interferon pathway during Mtb infection. Next, PI/Hoechst Double Staining was used to test the effects of UBE2L6 on cells. siRNA with the most significant interference effect (*p *= 0.024) was screened and act on cells(Figure 4A), and then, the cells were infected for 24 h, and further stained for observation. We found that compared with NC siRNA, apoptosis was significantly increased after UBE2L6 inhibition(*p *= 0.009), and there was no statistical difference in cell necrosis(Figure 4C). This suggests that UBE2L6 may promote the survival of intracellular Mtb.

### 3.6 Prediction and validation of miRNAs that may regulate UBE2L6

**FIGURE 5:**
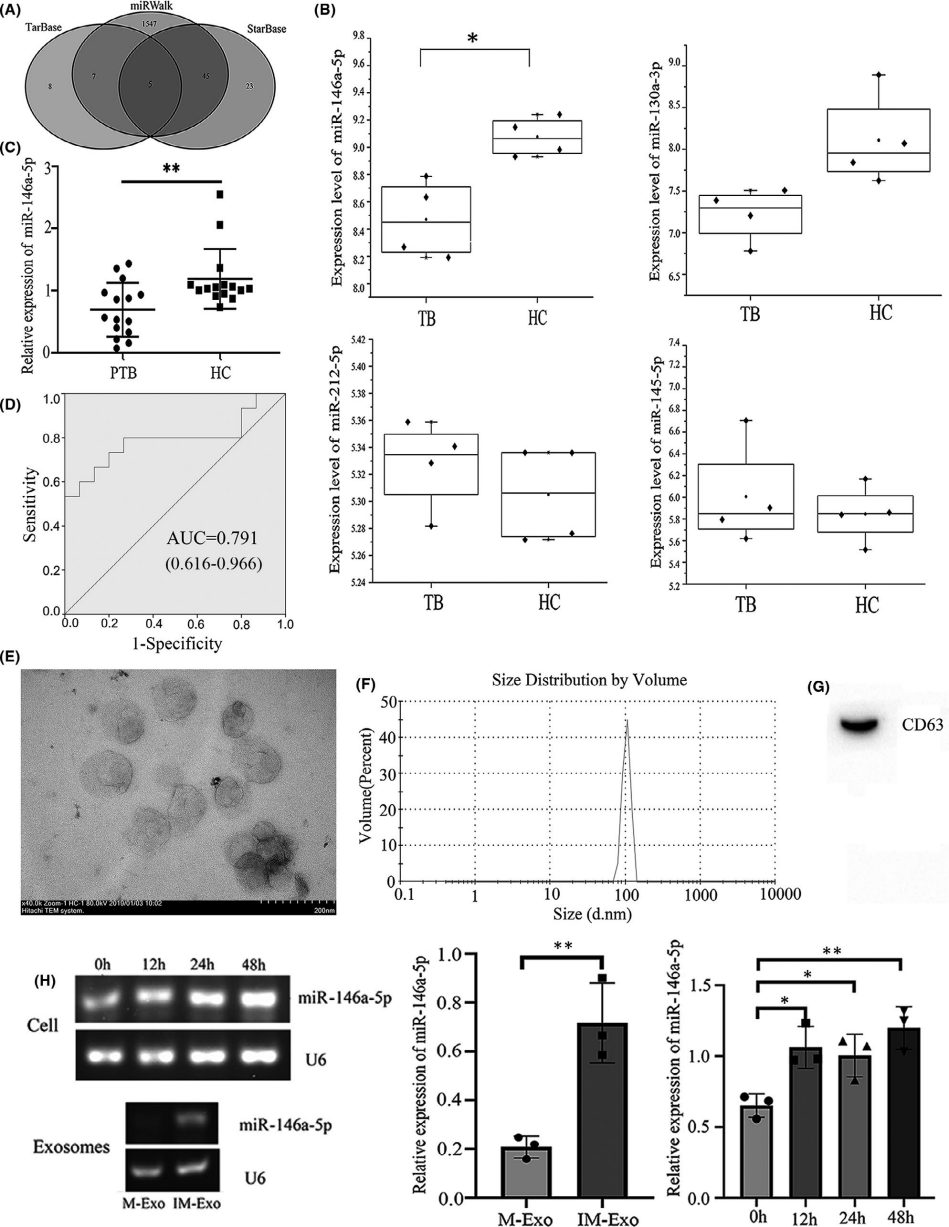
Predicted miRNAs and their validation. The number of predicted miRNAs by the StarBase, TarBase and miRWalk databases(A). Database validation of predicted miRNAs by GSE131708(B). Expression of miRNA‐146a‐5p in plasma of tuberculosis patients and healthy controls(C).ROC curve of miR‐146a‐5p in clinical plasmas collected(D).*:*p *< 0. 05 **:*p *< 0. 01***:*p *< 0. 001 TEM identification of exosome microstructure(E). NTA identification of exosome diameter abundance(F). WB identification of exosome signature protein CD63(G). Expression of miRNA‐146a‐5p in Mtb‐infected macrophages and exosomes from it(H)

miRNA is non‐coding regulatory small molecule that can play an important role by suppressing gene expression. We tried to find miRNA that interact with UBE2L6, so we used StarBase, TarBase and miRWalk to predict it, and the intersection of the results was the final predicted miRNAs (Figure 5A), they were namely miR‐145‐5p, miR‐212‐5p, miR‐1‐3p, miR‐130a‐3p and miR‐146a‐5p. We selected GSE131708 gene data set to verify the reliability of above miRNAs in TB. The statistical results (Figure 5B) indicated that compared with HC, miR‐146a‐5p was significantly reduced in PBMC of TB.

What about the expression of miR‐146a‐5p in plasma of TB patients? We collected plasma from 15 TB patients and 15 HC, and then, miR‐146a‐5p was quantitatively analysed by qRT‐PCR. The result indicated that the expression of miR‐146a‐5p was significantly low (*p *= 0.007) in the plasma of TB patients and was negatively correlated with UBE2L6(Figure 5C). At the same time, we also conducted ROC analysis on miRNA‐146a‐5p(Figure 5D), and the results showed that its AUC value, sensitivity and specificity were 0.791, 0.667 and 0.867 (95%CI:0.616–0.966). Specificity meets TPP's minimum standards, while sensitivity does not, which indicated that miRNA‐146a‐5p had mild clinical diagnostic value in TB.

We then examined miR‐146a‐5p in Mtb‐infected RAW264.7 cells and exosomes from it. First, the extracted exosomes were identified, and the results showed that a clear and typical goblet like vesicle structure of exosomes was observed by TEM, and the size was relatively uniform (Figure 5E). NTA showed that the diameter of exosomes was concentrated in the range of 80‐150nm, which was in line with the standard diameter of exosomes (Figure 5F). Western blot (WB) detected the exosome signature protein CD63(Figure 5G). In contrast to the above results, we found that miR‐146a‐5p increased significantly in Mtb‐infected RAW264.7 cells and exosomes from it (Figure 5H). It means that miR‐146a‐5p may indirectly affect the expression of UBE2l6 by influencing other molecules. The above results indicated that the expression of miR‐146a‐5p was significantly different in different cells during Mtb infection.

## 4 DISCUSSION

The End TB Strategy of the World Health Organization (WHO) aims to reduce the annual incidence of TB to less than 10 cases per 100,000 people by 2035.^14^ Therefore, it is urgent to accelerate the research on the mechanism of TB so as to carry out appropriate treatment and improve diagnostic methods to shorten the testing time, such as metabolic regulation mechanism in the host cell of Mtb and the molecular mechanism that affects the survival of bacteria in the cell when suffering from TB. Recently, researchers have attempted to find new biomarkers from the blood of TB patients for early detection, early diagnosis and development of effective treatment methods.^15^, ^16^ The molecules change during any disease, TB is the same. Such as formation of foamy macrophages by TB pleural effusions is triggered by the interleukin‐10/signal transducer and activator of transcription 3 Axis through ACAT upregulation.^17^, ^18^ In addition, Vrieling F found that oxidized low density lipoprotein supports the survival of Mtb in macrophages by inducing lysosomal dysfunction.^19^

In this study, we found that selected UBE2L6 is associated with protein ubiquitination and type I interferon in TB. Protein ubiquitination is an important cellular process targeting abnormal or short‐lived protein degradation and involves at least three types of enzymes: E1 ubiquitin activating enzyme, E2 ubiquitin conjugating enzyme and E3 ubiquitin ligase.^20^ Studies have revealed the unique mechanism of host xenophagocytosis triggered by the direct binding of ubiquitins to pathogen surface proteins.^21^ UBE2L6 gene encodes members of the ubiquitin‐binding enzyme family, it is required for the UV‐induced DNA damage response and ubiquitylation of Set8 and PCNA in addition to p21.^22^ Bioinformatics analysis showed that UBE2L6 is involved not only in protein ubiquitination but also in the innate immune system and in regulating cytokine production in Mtb‐infected THP‐1 cells. Our experiment also verified that type I interferon can induce UBE2L6 expression during Mtb infection. Consistent with above, several articles have elucidated the relationship between STATs and ubiquitin‐related enzymes, particularly in hypoxia, inflammation and tumour‐activated apoptosis. Studies have shown that the apoptosis and protein degradation induced by STAT1/2 requires ubiquitin‐related enzymes, including UBA7 and UBE2L6.^23^, ^24^, ^25^ Other studies have shown that UBE2L6 is involved in the lipolysis process in nasopharyngeal carcinoma and associated with its poor prognosis.^26^ The limitation of this study is that the changes of UBE2L6 in latent TB and different stages of active TB are not taken into account, which will be needed for further in‐depth research in the future.

UBE2L6 is both an E2 enzyme of ubiquitin and an E2 enzyme of ISG15. UBE2L6 has been confirmed by conjugated ISG15, and ISG15 has also been identified as a potential cancer serological marker.^27^, ^28^ Previous studies indicated that the expression of ISG15 and USP18 genes could be improved by type I interferon signalling pathway, and USP18, in turn, could play an inhibitory role in IFN signal transduction through STAT2. IFN‐α and ISG15 could also increase the expression of UBA7, UBE2L6 and HERC5 by activating STAT1 protein, and thus induced ISGylation of target proteins.^29^, ^30^, ^31^ Our bioinformatics analysis showed that ISG15 and USP18 were indeed upregulation in Mtb‐infected THP‐1 cells. In addition, some studies found that protein ISGylation was an antagonistic system of ubiquitylation.^32^, ^33^ Another study stated clearly that ISG15 and ubiquitin could form a mixed chain to regulate the stability of proteins and the expression of exogenous ISG15 increased the overall ubiquitylation pattern.^34^, ^35^ However, the interaction relationship between the above two in TB is not very clear and needs further study.

**FIGURE 6:**
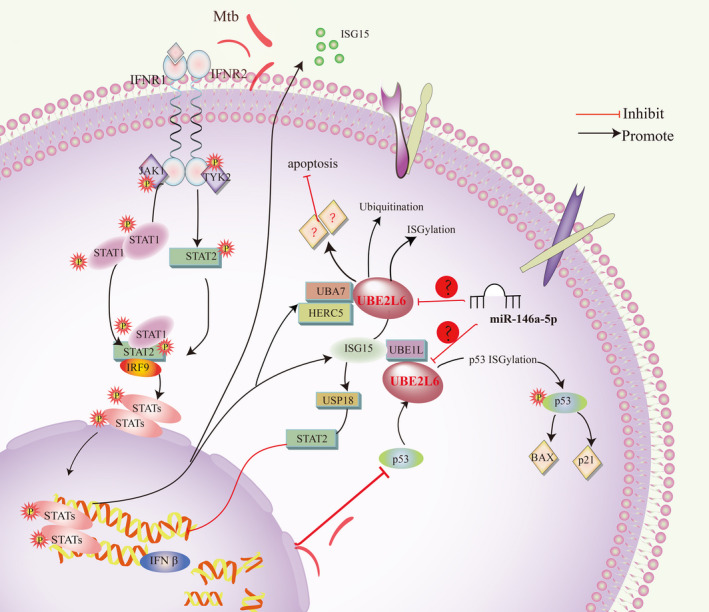
Intracellular mechanism pathway of UBE2L6

In addition to the type I interferon induced pathway, p53 can independently induce the expression of the ISG15 conjugation system, including UBE2L6, and it act as an endogenous target of ISGylation under conditions of DNA damage, further promoting cell growth inhibition and apoptosis by increasing its transactivity.^36^ In a study on cervical cancer, the authors found that UBE2L6,as a new target of UHRF1, can regulate the apoptosis function of UHRF1.^37^ In this analysis, we found that the expression of UHRF1 did not change significantly compared with control group, but this does not mean that it is not related to UBE2L6. It is possible that modifications or structural changes of UHRF1 may lead to changes of UBE2L6, which need to be identified. What is inconsistent with the above studies is that our results show that UBE2L6 inhibits cell apoptosis during Mtb infection, but has no effect on cell necrosis. How UBE2L6 affects apoptosis still needs to be further studied. In view of previous research conclusions, bioinformatics analysis and our experiments, we can infer the molecular mechanism of UEB2L6 in TB, as shown in the Figure 6 below. First of all, UBE2L6 plays the role of ubiquitin‐binding enzyme in protein ubiquitination. Secondly, infection of Mtb activated the type 1 interferon pathway, leading to increased expression of the ISG15 conjugate system, including UBE2L6 and further leading to ISGylation of the target protein, and then, the exocrine secretion of ISG15 stimulates lymphocytes and monocytes to produce IFN‐γ. The increase of UBE2L6 in Mtb infection also causes changes in downstream molecules, which inhibit cell apoptosis, and this will have a great impact on the survival of Mtb in cells. Moreover, the decrease of p53 also inhibit the expression of UBE2L6 and ISG15, and further suppress p53 ISGylation, inhibiting the expression of its downstream targets, such as BAX and P21.

Studies have shown that the expression of miR‐146a in PBMC of TB patients is downregulated.^38^ It has also been found that miR‐146a attenuates the production of TNF‐α in BCG‐induced THP‐1 cells and promotes the survival of BCG by inhibiting the production of NO in macrophages.^39^, ^40^ Thus, miR‐146a plays an important role during Mtb infection. Our verification results also showed that miR‐146a‐5p was declined in PBMC and plasma of TB, and it is negatively correlated with UBE2L6, suggesting that miR‐146a‐5p may participate in protein metabolism and innate immunity by regulating UBE2L6, but the specific mechanism needs to be further investigated. However, they are expressed in the same direction in the Mtb‐infected RAW264.7 cell line, suggesting that miR‐146a‐5p is not involved in the regulation of UBE2L6 in RAW264.7 cells or there are other unknown and complex regulatory mechanisms, which need to be further investigated. It is regrettable that we have not experimented to verify the regulatory relationship between UBE2L6 and miR‐146a‐5p.

In conclusion, we found that UBE2L6 was significantly upregulated in blood of TB patients, Mtb‐infected THP‐1cells and RAW264.7 cells. This may means that the increase of intracellular UBE2L6 and then secreted extracellular with exosomes or by other means. Subsequent experiments are needed to test this idea. In addition to this, it also had high clinical diagnostic value. The significant upregulation of UBE2L6 in Mtb infection may be caused by two mechanisms. Firstly, the expression of UBE2L6 was increased by activating type Ⅰ interferon pathway during Mtb infection, which has been confirmed. Secondly, the downregulation of miR‐146a‐5p during Mtb infection weakened its inhibitory effect on UBE2L6, thus increasing the expression of UBE2L6. The prediction and identification of different biological effects of UBE2L6 can provide reference for further study of the molecular mechanism of TB.

## CONFLICT OF INTEREST

All authors: no reported conflicts.

## AUTHOR CONTRIBUTIONS

Jiao Gao designed the study and wrote the paper. Chonghui Li: collected and processed the clinical sample. Wenjing Li and Haotian Chen conducted cell and exosome‐related experiments. Jiao Gao, Chonghui Li and Haotian Chen analysed the data. Yurong Fu and Zhengjun Yi involved in technical guidance and research funding support.

## Data Availability

The data sets used for the current study are available from the corresponding author upon reasonable request.
